# Prognostic role of prognostic nutritional index in patients with bladder cancer: a systematic review and meta-analysis

**DOI:** 10.3389/fonc.2024.1486389

**Published:** 2025-01-23

**Authors:** Jing Sun, Zhenzhen Li, Xiaming Zhu

**Affiliations:** The Affiliated Taizhou People’s Hospital of Nanjing Medical University, Taizhou, China

**Keywords:** prognostic nutritional index, bladder cancer, overall survival, recurrence-free survival, meta-analysis

## Abstract

**Aims:**

An increasing number of studies have explored the prognostic significance of the prognostic nutritional index (PNI) in bladder cancer patients, but the results are inconsistent. This study systematically investigates the prognostic value of baseline PNI in patients with bladder cancer through a meta-analytic approach.

**Methods:**

The databases of PubMed, EmBase, and the Cochrane library were systematically searched for eligible studies from inception until April 2024. The prognostic outcomes including overall survival (OS) and recurrence-free survival (RFS). The summary outcomes were calculated using the random-effects model, and the exploratory analyses were performed by sensitivity and subgroup analyses.

**Results:**

Twelve retrospective studies involved 2,951 patients with bladder cancer were selected in final analysis. The summary results found low PNI were associated with poor OS (HR: 1.80; 95%CI: 1.54-2.10; *P*<0.001) and RFS (HR: 1.53; 95%CI: 1.15-2.04; *P*=0.003). The association between low PNI and shorter OS was statistically significant in all subgroups. Additionally, the association between low PNI and RFS was also significant in most subgroups.

**Conclusions:**

This study found a significant association between low PNI and poor prognosis in bladder cancer patients. Further large-scale prospective study should be performed to verify this association, and assess the nutrition interventions for patients with bladder cancer.

**Systematic review registration:**

https://inplasy.com/inplasy-2024-8-0020/, identifier INPLASY202480020.

## Introduction

Bladder cancer is one of the most common malignant tumors of the urinary system, originating from the urothelium of the bladder (1). According to the 2020 global cancer statistics, bladder cancer ranks ninth in terms of incidence and thirteenth in terms of mortality among all cancers (2). More than half of bladder cancer cases occur in high-income countries, with the highest incidence rates in North America and Europe, and the lowest in Africa (2). The recurrence rate of bladder cancer is relatively high, with a 5-year recurrence rate of approximately 65% for non-invasive or *in situ* tumors at the initial diagnosis, and 73% for patients diagnosed at a slightly later stage of the disease (3). Therefore, bladder cancer ranks as the most expensive malignant tumor to treat among all cancers, with estimated costs per patient ranging from $89,287 to $202,203, imposing a significant economic burden on society and families (4, 5).

Transurethral resection of bladder tumor (TURBT) is the main treatment for non-muscle-invasive bladder cancer, but for muscle-invasive bladder cancer, radical cystectomy and urinary diversion combined with neoadjuvant chemotherapy are the main treatment methods (6). Given the poor prognosis of bladder cancer, it is particularly important to explore robust and accurate prognostic factors to predict the outcome of bladder cancer. Numerous studies have shown the association between nutritional status and cancer prognosis (7–10). Patient’s nutritional status can affect immune function, treatment tolerance, postoperative recovery, and quality of life, thereby influencing patient prognosis (11). The prognostic nutritional index (PNI) is an indicator used to assess the nutritional status of surgical patients preoperatively by utilizing serum albumin levels and peripheral blood lymphocyte counts, with the aim of preliminarily predicting the probability of postoperative complications in surgical patients (12). While the prognostic value of the PNI for bladder cancer patients has been extensively investigated, the findings remain controversial (13–24). This study aims to systematically evaluate the prognostic value of PNI for bladder cancer patients by using a meta-analytic approach.

## Methods

### Data sources, search strategy, and selection criteria

This review follows the requirements and reporting guidelines of the Preferred Reporting Items for Systematic Reviews and Meta-Analyses (PRISMA) statement (25). This study was registered in INPLASY platform (no: INPLASY202480020). Any study investigating the association of PNI with the prognosis of bladder cancer met the inclusion criteria, with no restrictions on publication language or status. Literature search was conducted on electronic databases including PubMed, Embase, and Cochrane Library up to April 2024. The search terms included “prognostic nutritional index” or “PNI” and “bladder cancer” and “human”. We also manually searched the reference lists of all relevant original and review articles to identify any additional studies meeting the inclusion criteria.

Literature search and study selection was independently conducted by two authors using standardized methods. In cases of disagreement, consensus was reached through mutual discussion. Studies were included if they met the following inclusion criteria: (1) Patients: all of patients diagnosed with bladder cancer; (2) Exposure: low PNI; (3) Control: high PNI; (4) Outcomes: overall survival (OS) and recurrence-free survival (RFS); and (5) Study design: prospective or retrospective design.

### Data collection and quality assessment

The collected data included the surname of the first author, publication year, study design, country, sample size, mean age, male proportion, pT3-4 proportion, high grade proportion, disease status, treatments, cutoff value of PNI and assessments, malnourished proportion, duration of follow-up, estimated effect size, and its 95% confidence interval (CI). For studies reporting several multivariable adjusted effect sizes, we selected the effect estimate with the maximum adjustment for potential confounders. Methodological quality assessment was conducted using the Newcastle-Ottawa Scale (NOS), which comprises components related to selection (4 items), comparability (1 item), and outcome (3 items) (26). Each study was scored on a scale of 0-9. Data extraction and quality assessment were independently performed by two authors, with any discrepancies resolved through consultation with another author who reviewed the original studies independently.

### Statistical analysis

We analyzed the relationship between PNI and bladder cancer prognosis based on the reported effect estimates and their 95% CI in each study. For OS and RFS, we assessed the effect estimates using hazard ratios (HR) and 95%CI. All of pooled analyses were calculated using the random-effects model, which considering the underlying varies across included studies (27, 28). The heterogeneity was assessed using *I^2^* and Q statistic, and the significant heterogeneity was defined by *I^2^* ≥ 50.0% or *P* < 0.10 (29, 30). Sensitivity analyses were performed to assess the stability of pooled conclusion by sequential removing single study (31). The sources of heterogeneity in estimates of the association of PMI with OS and RFS were explored by using univariate meta-regression analysis (32). Subgroup analyses were also performed according to country, sample size, mean age, male proportion, treatments, cutoff value, cutoff value determination, follow-up, or study quality, and the differences between subgroups were compared using the interaction *t* test, which assuming the data met normal distribution (33). Publication bias for OS and RFS were assessed using funnel plots, Egger, and Begg tests (34, 35). All reported *P* values were two-sided, and a *P* value < 0.05 for the combined results was considered statistically significant. STATA software (version 12.0; Stata Corp, College Station, TX, USA) was used for analysis.

## Results

### Literature search

**Figure 1:**
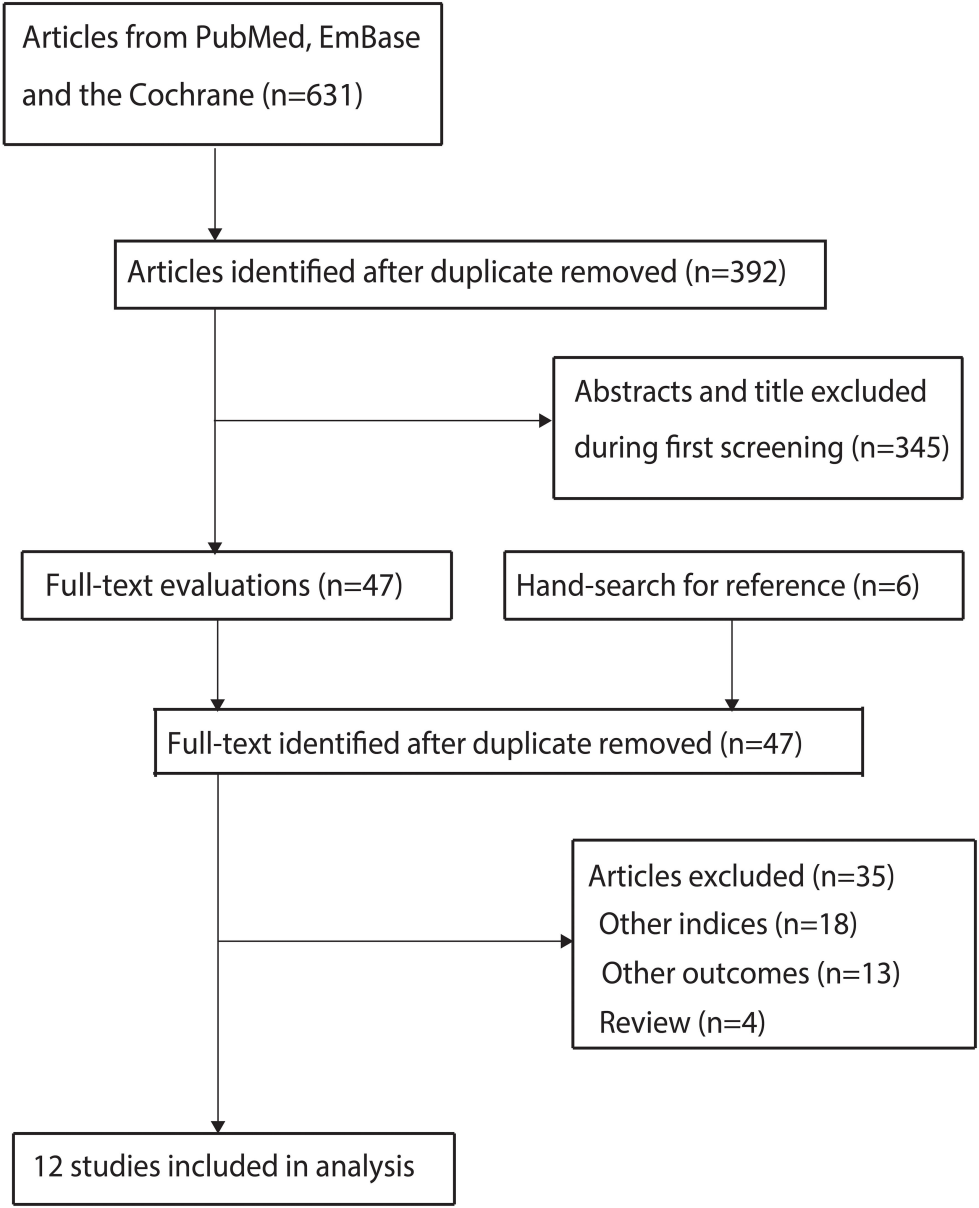
The PRISMAR flowchart for literature search and study selection.

A total of 631 articles were identified from initial electronic searches, and 392 articles were retained after duplicate articles were removed. Through title and abstract screening, we further excluded 345 studies. We conducted full-text retrieval for the remaining 47 studies, resulting in the exclusion of 35 studies because of: other nutritional indices (n=18), reported other outcomes (n=13), and review (n=4). Manual search did not reveal any new studies that met the inclusion criteria. Finally, a total of 12 studies were selected for meta-analysis (13–24), and the details regarding literature search and study selection is shown in **Figure 1**.

### Study characteristics

The baseline characteristics of identified studies and involved patients is summarized in **Table 1**. All of included studies were retrospective cohort design, and a total of 2,951 patients with bladder cancer were included. Eight studies were conducted in China or Japan, and the remaining 4 studies were conducted in Turkey, Austria, or Spain. The sample size ranged from 68-516, while the follow-up duration ranged 13.5-108.0 months. In the studies selected, the PNI cutoff values ranged from 40.00 to 52.57. This range highlights the variability in the PNI thresholds used across different studies, which is an important consideration when interpreting the results. Nine studies included patients received radical cystectomy, 2 studies included patients treated with TURBY, and the remaining 1 study included patients treated with radiotherapy. Study quality was assessed in NOS, 4 studies with 8 stars, 4 studies with 7 stars, and the remaining 4 studies with 6 stars.

**Table 1:** The baseline characteristics of included studies and involved patients.

Study	Study design	Country	Sample size	Age (years)	Male (%)	pT stage (1/2/3/4)	High grade (%)	Disease status	Treatment	Cutoff	Malnourished (%)	Follow-up (months)	NOS
Peng 2017 (13)	Retrospective	China	516	66.0	84.5	162/161/105/88	NA	BC	Radical cystectomy	46.0/47.0 (ROC)	NA	37.0	8
Miyake 2017 (14)	Retrospective	Japan	117	72.0	78.0	0/60/29/28	NA	Muscle-invasive BC	Radical cystectomy	50.1 (median)	NA	22.0	8
Cui 2017 (15)	Retrospective	China	329	62.9	79.6	82/0/0/0	25.8	Non-muscle-invasive BC	TURBT	52.57 (ROC)	51.4	43.9	8
Zhu 2019 (16)	Retrospective	China	186	65.0	84.4	39/125/22	48.4	BC patients undergoing robotic surgery	Radical cystectomy	50.95 (ROC)	NA	20.6	6
Stangl-Kremser 2019 (17)	Retrospective	Austria	68	82.0	80.9	6/56/5/1	NA	Urothelia carcinoma of the bladder	Radiotherapy	45.2 (median)	NA	13.5	6
Yilmaz 2020 (18)	Retrospective	Turkey	152	66.0	87.5	0/99/41/12	63.8	Muscle-invasive BC	Radical cystectomy	45.9 (ROC)	NA	16.0	7
Bi 2020 (19)	Retrospective	China	387	69.5	71.6	260/0/0/0	67.9	Non-muscle-invasive BC	TURBT	50.17 (ROC)	29.9	108.0	8
Wang 2023 (20)	Retrospective	China	262	66.0	90.1	0/124/112/26	58.8	Muscle-invasive BC	Radical cystectomy	47.7 (K-M)	NA	32.0	6
Zhong 2023 (21)	Retrospective	China	373	65.3	86.9	0/172/118/47	NA	Muscle-invasive BC	Radical cystectomy	45.0	NA	46.0	7
Teke 2023 (22)	Retrospective	Turkey	173	64.3	85.5	0/93/57/23	NA	Urothelia carcinoma of the bladder	Radical cystectomy	47.0 (ROC)	NA	21.0	7
Moreno-Cortes 2023 (23)	Retrospective	Spain	294	72.0	87.4	51/12/91/72	61.2	Muscle-invasive BC	Radical cystectomy	40.0	NA	50.0	7
Zhang 2024 (24)	Retrospective	China	94	71.6	89.4	0/61/33/0	77.7	Muscle-invasive BC	Radical cystectomy	44.15 (ROC)	NA	15.3	6

*BC, bladder cancer; K-M, Kaplan-Meier survival analysis; ROC, receiver operating characteristics; TURBT, transurethral resection of bladder tumor.

### Overall survival

**Figure 2:**
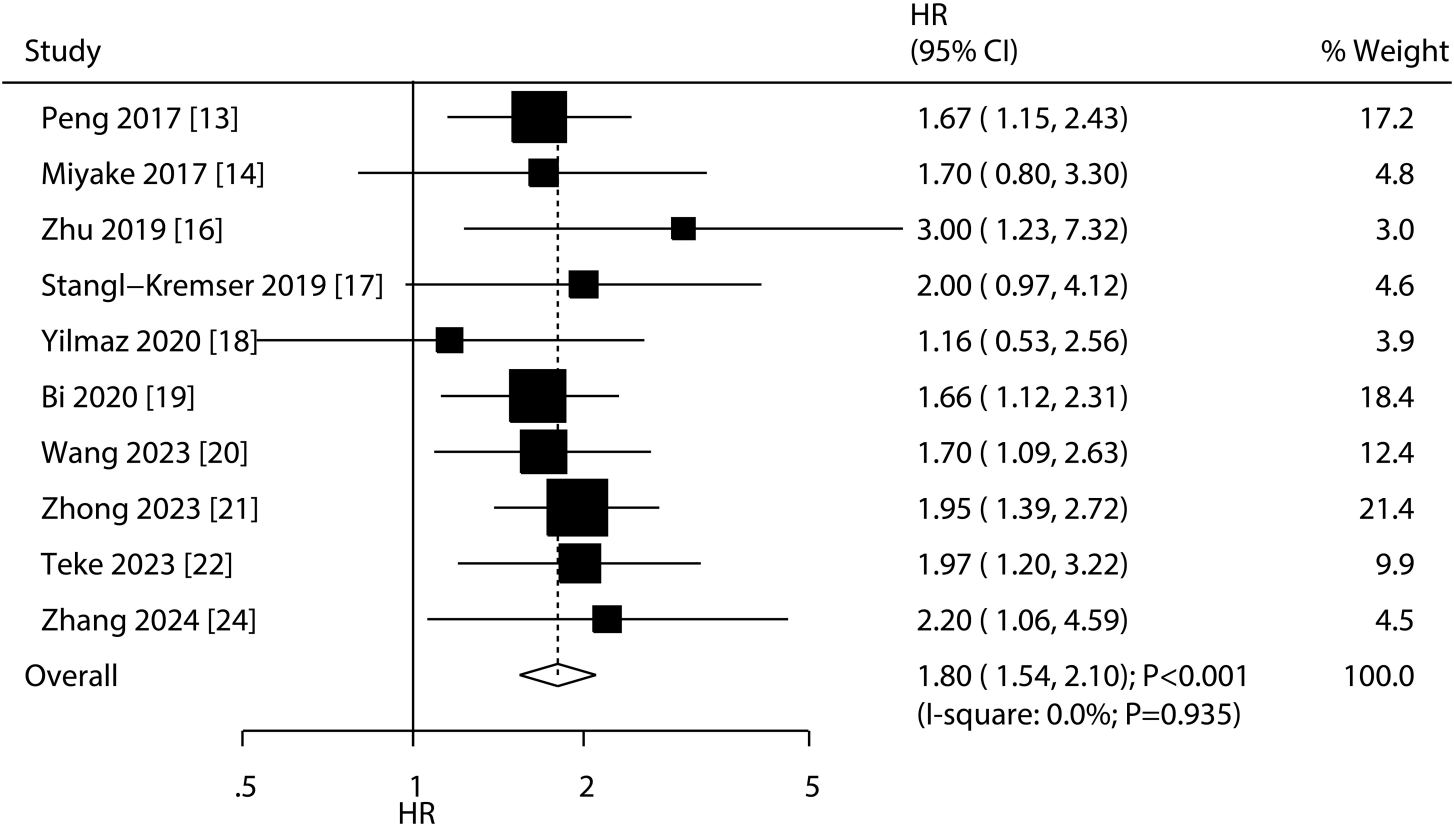
Association of low PNI with OS in patients with bladder cancer.

A total of 10 studies reported the association of PNI with OS, and the summary result indicated low PNI was associated with poor OS as compared with high PNI (HR: 1.80; 95%CI: 1.54-2.10; *P*<0.001; **Figure 2**). There was no evidence of heterogeneity among included studies (*I^2^ =* 0.0%; *P*=0.935). Sensitivity analysis indicated that the pooled conclusion was robust and remained unchanged when sequential excluding each individual study (**Supplementary Figure S1**). Meta-regression analyses found country (*P*=0.911), sample size (*P*=0.618), mean age (*P*=0.687), male proportion (*P*=0.595), treatments (*P*=0.760), cutoff value (*P*=0.947), cutoff value determination (*P*=0.763), follow-up (*P*=0.778), and study quality (*P*=0.517) were not contributed a significant role regarding the association of PNI with OS (**Table 2**). Subgroup analyses found that low PNI was associated with poor OS in all subsets, and the differences between subgroups were not statistically significant (**Table 2**).

**Table 2:** Subgroup analyses for OS and RFS.

Outcomes	Factors	Subgroups	HR and 95%CI	*P* value	*I^2^* (%)	Q statistic	Meta-regression	Ratio of HR between subgroups
OS	Country	China or Japan	1.81 (1.52-2.15)	< 0.001	0.0	0.900	0.911	1.02 (0.68-1.53); *P*=0.913
		Turkey or Europe	1.77 (1.23-2.54)	0.002	0.0	0.497		
	Sample size	≥ 200	1.75 (1.46-2.11)	< 0.001	0.0	0.906	0.618	0.92 (0.65-1.28); *P*=0.610
		< 200	1.91 (1.44-2.53)	< 0.001	0.0	0.730		
	Mean age (years)	≥ 70	1.95 (1.29-2.96)	0.002	0.0	0.881	0.687	1.10 (0.70-1.71); *P*=0.690
		< 70	1.78 (1.50-2.10)	< 0.001	0.0	0.784		
	Male proportion (%)	≥ 80	1.84 (1.54-2.20)	< 0.001	0.0	0.854	0.595	1.10 (0.76-1.59); *P*=0.605
		< 80	1.67 (1.21-2.30)	0.002	0.0	0.953		
	Treatments	Radical cystectomy	1.83 (1.53-2.18)	< 0.001	0.0	0.855	0.760	1.06 (0.74-1.54); *P*=0.741
		Other	1.72 (1.25-2.38)	0.001	0.0	0.652		
	Cutoff value	≥ 50	1.79 (1.32-2.42)	< 0.001	0.0	0.478	0.947	0.99 (0.70-1.41); *P*=0.951
		< 50	1.81 (1.51-2.16)	< 0.001	0.0	0.907		
	Cutoff determination	ROC	1.76 (1.44-2.17)	< 0.001	0.0	0.672	0.763	0.95 (0.70-1.30); *P*=0.754
		Other	1.85 (1.46-2.34)	< 0.001	0.0	0.953		
	Follow-up (months)	≥ 36	1.77 (1.44-2.17)	< 0.001	0.0	0.766	0.778	0.96 (0.70-1.31); *P*=0.782
		< 36	1.85 (1.46-2.34)	< 0.001	0.0	0.809		
	Study quality	High	1.75 (1.46-2.09)	< 0.001	0.0	0.874	0.517	0.89 (0.62-1.28); *P*=0.521
		Moderate	1.97 (1.44-2.70)	< 0.001	0.0	0.712		
RFS	Country	China or Japan	1.81 (1.48-2.22)	< 0.001	0.0	0.449	0.007	1.57 (0.88-2.81); *P*=0.124
		Turkey or Europe	1.15 (0.67-1.98)	0.611	80.9	0.005		
	Sample size	≥ 200	1.75 (1.44-2.13)	< 0.001	0.0	0.908	0.016	1.34 (0.63-2.81); *P*=0.446
		< 200	1.31 (0.64-2.69)	0.456	85.8	0.001		
	Mean age (years)	≥ 70	2.24 (1.26-3.99)	0.006	30.4	0.231	0.093	1.61 (0.83-3.11); *P*=0.156
		< 70	1.39 (1.01-1.91)	0.041	75.3	0.003		
	Male proportion (%)	≥ 80	1.28 (0.87-1.88)	0.210	77.5	0.004	0.027	0.66 (0.40-1.07); *P*=0.089
		< 80	1.95 (1.45-2.61)	< 0.001	9.6	0.331		
	Treatments	Radical cystectomy	1.44 (0.97-2.12)	0.069	77.4	0.001	0.122	0.80 (0.49-1.30); *P*=0.358
		Other	1.81 (1.35-2.42)	< 0.001	0.0	0.516		
	Cutoff value	≥ 50	1.95 (1.45-2.61)	< 0.001	9.6	0.331	0.027	1.52 (0.94-2.47); *P*=0.089
		< 50	1.28 (0.87-1.88)	0.210	77.5	0.004		
	Cutoff determination	ROC	1.39 (1.01-1.91)	0.041	75.3	0.003	0.093	0.62 (0.32-1.20); *P*=0.156
		Other	2.24 (1.26-3.99)	0.006	30.4	0.231		
	Follow-up (months)	≥ 36	1.75 (1.44-2.13)	< 0.001	0.0	0.908	0.016	1.34 (0.63-2.81); *P*=0.446
		< 36	1.31 (0.64-2.69)	0.456	85.8	0.001		
	Study quality	High	1.53 (1.15-2.04)	0.003	70.7	0.002		–
		Moderate	–	–	–	–		

### Recurrence-free survival

**Figure 3:**
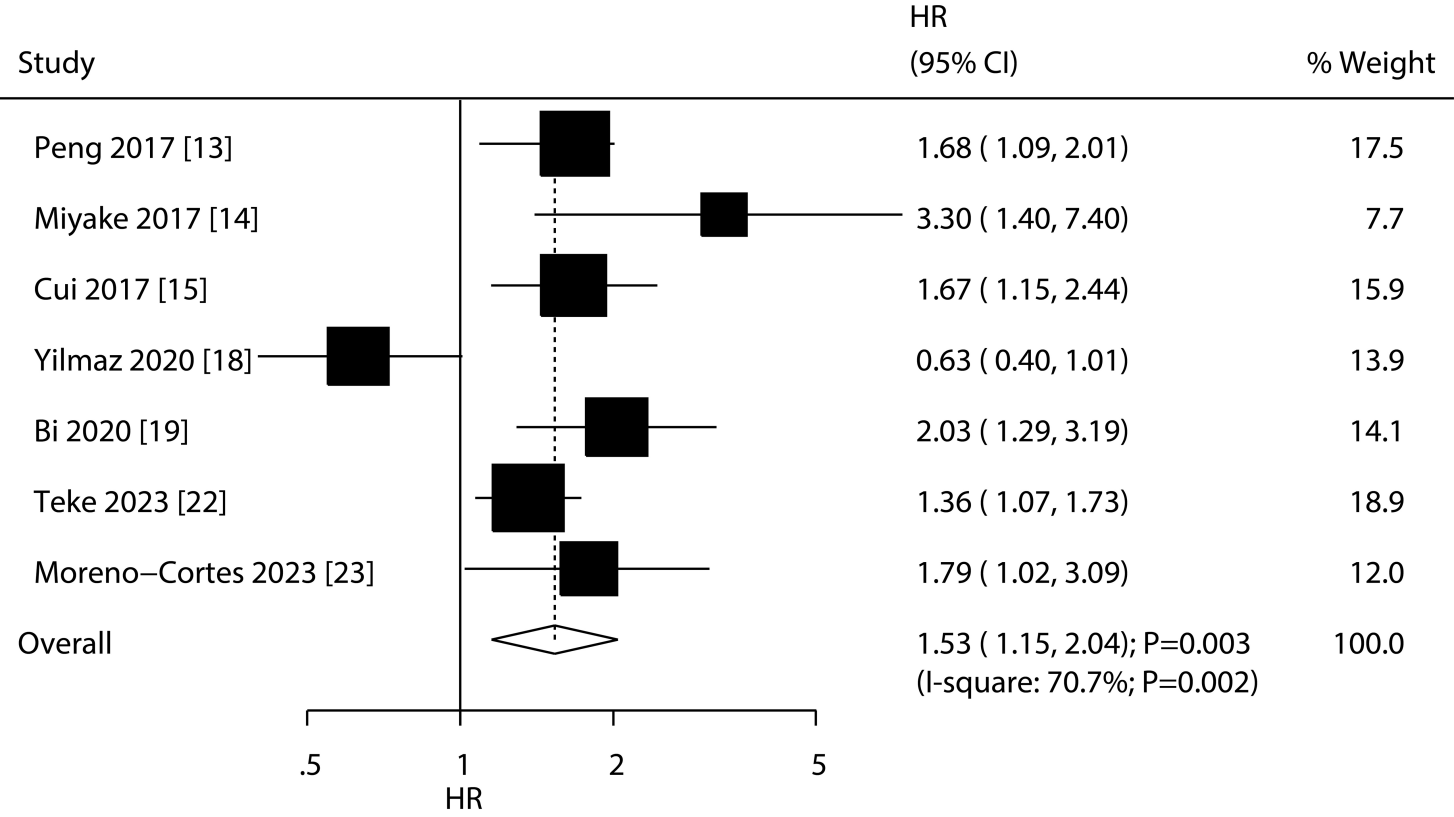
Association of low PNI with RFS in patients with bladder cancer.

A total of 7 studies reported the association of PNI with RFS, and the summary result indicated low PNI was associated with poor RFS as compared with high PNI (HR: 1.53; 95%CI: 1.15-2.04; *P*=0.003; **Figure 3**). There was significant heterogeneity across included studies (*I^2^ =* 70.7%; *P*=0.002). After removing study conducted by Yilmaz et al., the heterogeneity was reduced (*I^2^ =* 16.7%; *P*=0.306), and the pooled HR was 1.65 (95%CI: 1.39-1.96; *P*<0.001; **Supplementary Figure S2**). The meta-regression analysis found country (*P*=0.007), sample size (*P*=0.016), male proportion (*P*=0.027), cutoff value (*P*=0.027), and follow-up (*P*=0.016) contributed to the association between PNI and RFS (**Table 2**). Although low PNI was associated with poor RFS in mostly subgroups, we noted low PNI was not associated with RFS if pooled studies conducted in Turkey or Europe, sample size < 200, male proportion ≥ 80.0%, patients treated with radical cystectomy, cutoff value < 50.0, and follow-up < 36.0 months (**Table 2**).

### Publication bias

**Figure 4:**
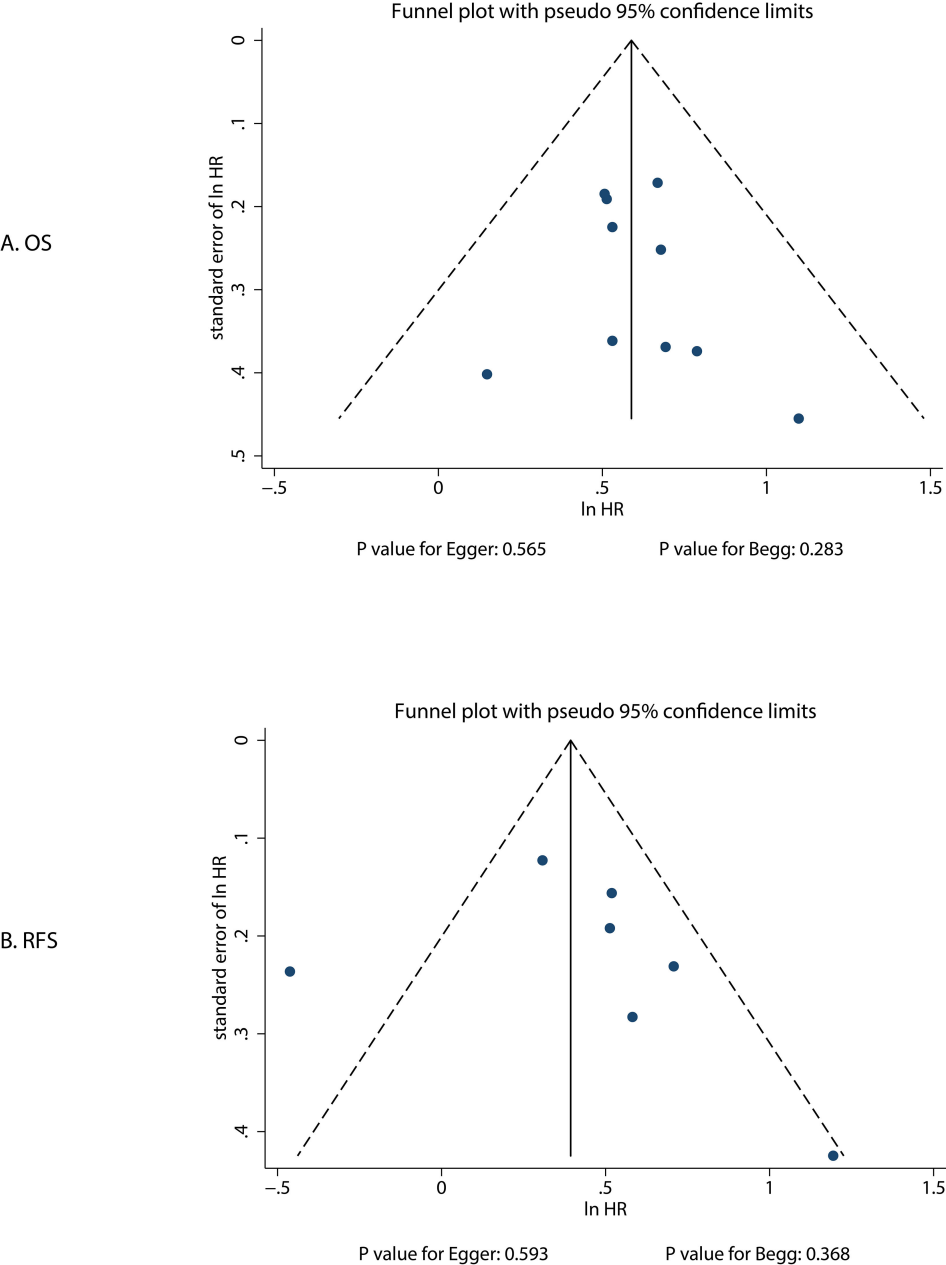
Funnel plots for OS and RFS.

We assessed publication bias for OS and RFS, and the results showed that the distribution of studies in the funnel plot was relatively symmetrical (**Figure 4**). Additionally, quantitative analysis indicated no significant publication bias regarding the association between PNI and OS (*P* value for Egger: 0.565; *P* value for Begg: 0.283) and RFS (*P* value for Egger: 0.593; *P* value for Begg: 0.368).

## Discussion

This study, for the first time, explored the role of PNI in the prognosis of bladder cancer patients using a meta-analytic approach. Our systematic search identified 12 studies, comprising 2,951 bladder cancer patients, that met the inclusion criteria. The severity of patients’ conditions and their baseline characteristics varied widely across the included studies. All included studies were of moderate to high quality, and the conclusions drawn from these studies hold significant clinical value. This study found low PNI was associated with a poor OS, and this conclusion was not affected by patients’ characteristics. Moreover, low PNI was associated with poor RFS, and this significant association mainly observed in studies conducted in China or Japan, sample size ≥ 200, irrespective mean age of patients or cutoff value determination, male proportion < 80.0%, patients received other treatments, cutoff value of PNI ≥ 50, and follow-up duration ≥ 36.0 months.

In previous study, the role of different nutritional indices on the prognosis of bladder cancer has been explored, with a total of 13 studies meeting the criteria. The results revealed a significant association between low PNI and poorer OS; however, there was no significant association between preoperative PNI and RFS (36). However, this study has the following limitations: (1) the study included only 7 studies, and some of the data extracted for analysis were erroneous; (2) subgroup analysis for OS in the study included only 5 factors, while other factors were not explored; and (3) there was a lack of exploratory analysis for PNI and RFS. Given that the latest published studies in recent years were not included in previous study (36), this study thoroughly investigates the impact of PNI levels on postoperative OS and RFS in patients with bladder cancer.

The summary result found low PNI was associated with a poor OS as compared with high PNI, and this conclusion was persistent in exploratory analyses, including sensitivity and subgroup analyses. The potential reasons for this significant association could explained by: (1) impaired immune function: low PNI reflects the nutritional status and immune status of patients, and the weakening of immune function may lead to evasion and resistance of cancer cells to immune surveillance, thereby promoting tumor development and metastasis, and affecting patient survival rates (37); (2) decreased treatment tolerance: malnutrition may lead to decreased tolerance to cancer treatment, including surgery, chemotherapy, and radiotherapy, thereby affecting treatment efficacy and patient survival (38); and (3) restricted postoperative recovery: low PNI may limit the recovery process after surgery, prolonging the patient’s recovery time, increasing the incidence of postoperative complications, and thereby affecting OS (39).

We noted low PNI was associated with poor RFS for patients with bladder cancer. Low PNI reflects poor nutritional status and weakened immune function in patients, which may reduce postoperative surveillance and clearance of residual tumor cells, thereby promoting tumor recurrence (40). Additionally, low PNI can affect wound healing and increase the risk of postoperative complications. The presence of delayed wound healing and complications creates a favorable environment for tumor recurrence (41). Furthermore, malnutrition can influence the tumor microenvironment, creating favorable conditions for tumor recurrence. Changes in the tumor microenvironment, such as increased inflammation and angiogenesis, may facilitate the growth and survival of residual tumor cells postoperatively (42). Finally, malnutrition may impair DNA repair mechanisms and cellular functions, increasing genomic instability and the likelihood of tumor cell survival and proliferation, thereby promoting recurrence (43).

Subgroup analyses found low PNI was associated with a poor RFS in mostly subgroups, whereas no significant association between PNI and RFS if pooled studies conducted in Turkey or Europe, sample size < 200, male proportion ≥ 80.0%, patients treated with radical cystectomy, cutoff value < 50.0, and follow-up < 36.0 months. The main reasons for this outcome are the number of studies included in the subgroup and the frequency of recurrence events, which affect the statistical power of the studies. Therefore, this conclusion needs to be validated by future large-scale prospective studies.

Compared with other nutritional indices, PNI has several advantages in the contest of bladder cancer. Unlike more complex and resource-intensive measures, PNI is derived from two readily available and inexpensive parameters: serum albumin levels and peripheral blood lymphocyte counts (12). This simplicity makes PNI easy to calculate and integrate into routine clinical practice without additional costs or specialized equipment. Additionally, PNI has been shown to have a stable and significant association with both OS and RFS, making it a robust and reliable prognostic tool. Its ability to provide valuable prognostic information with minimal effort and cost sets it apart from other indices, which may require more extensive data collection and analysis. The significant association between PNI and OS and RFS highlights the potential of PNI as a robust prognostic marker. Incorporating PNI into routine clinical practice can aid in risk stratification, allowing clinicians to identify high-risk patients who may benefit from more aggressive or tailored treatment strategies. Moreover, clinicians can use PNI to provide patients with more accurate and personalized information about their prognosis, helping them make informed decisions about their treatment options. In addition, previous studies have demonstrated that nutritional supplementation can increase the number and activity of immune cells, thereby enhancing the body’s immune defense capabilities. It also regulates the production of inflammatory factors, reducing excessive inflammatory responses and minimizing tissue damage. Consequently, this leads to a reduction in the incidence of perioperative and postoperative complications (44–46). Therefore, PNI should be integrated into electronic health records, and clinical guidelines and protocols should be developed for its use.

This study has the following limitations. Firstly, all included studies were retrospective in design, which may be subject to recall bias and uncontrollable confounding bias. Secondly, the heterogeneity for RFS were not fully explanation by sensitivity and subgroup analyses. Thirdly, there is inconsistency in the cutoff values and methods used to determine the cutoff values for PNI index, which may affect the magnitude of the association between PNI and bladder cancer prognosis. Fourthly, variations in disease severity (tumor grade and stage) and treatment modalities among included patients may significantly influence the prognosis of bladder cancer patients (47–51). Fifthly, mostly included studies were conducted on Asian populations (8/12 studies from China/Japan), which restricted the generalizability of the findings. Lastly, inherent limitations of meta-analysis based on published studies include inevitable publication bias and the inability to conduct in-depth exploratory analysis as the study analyses are based on pooled data.

## Conclusions

This study found a significant association between low PNI and shorter OS and RFS in bladder cancer patients. Exploratory analysis results indicated a relatively stable degree of association between PNI and prognosis of bladder cancer. While our study provides compelling evidence for the prognostic value of PNI in bladder cancer, further research is needed to validate these findings and to assess the association between PNI and the risk of treatment-related complications. Additionally, the long-term economic impact of PNI-based interventions for patients with bladder cancer should be explored.

## Data Availability

The original contributions presented in the study are included in the article/**Supplementary Material**. Further inquiries can be directed to the corresponding author.
