# Supplementary figures and images for: Prognostic role of prognostic nutritional index in patients with bladder cancer: a systematic review and meta-analysis
Source: Front Oncol. 2025 Jan 23;14:1486389. doi: 10.3389/fonc.2024.1486389 (PMC11798790; doi:10.3389/fonc.2024.1486389)

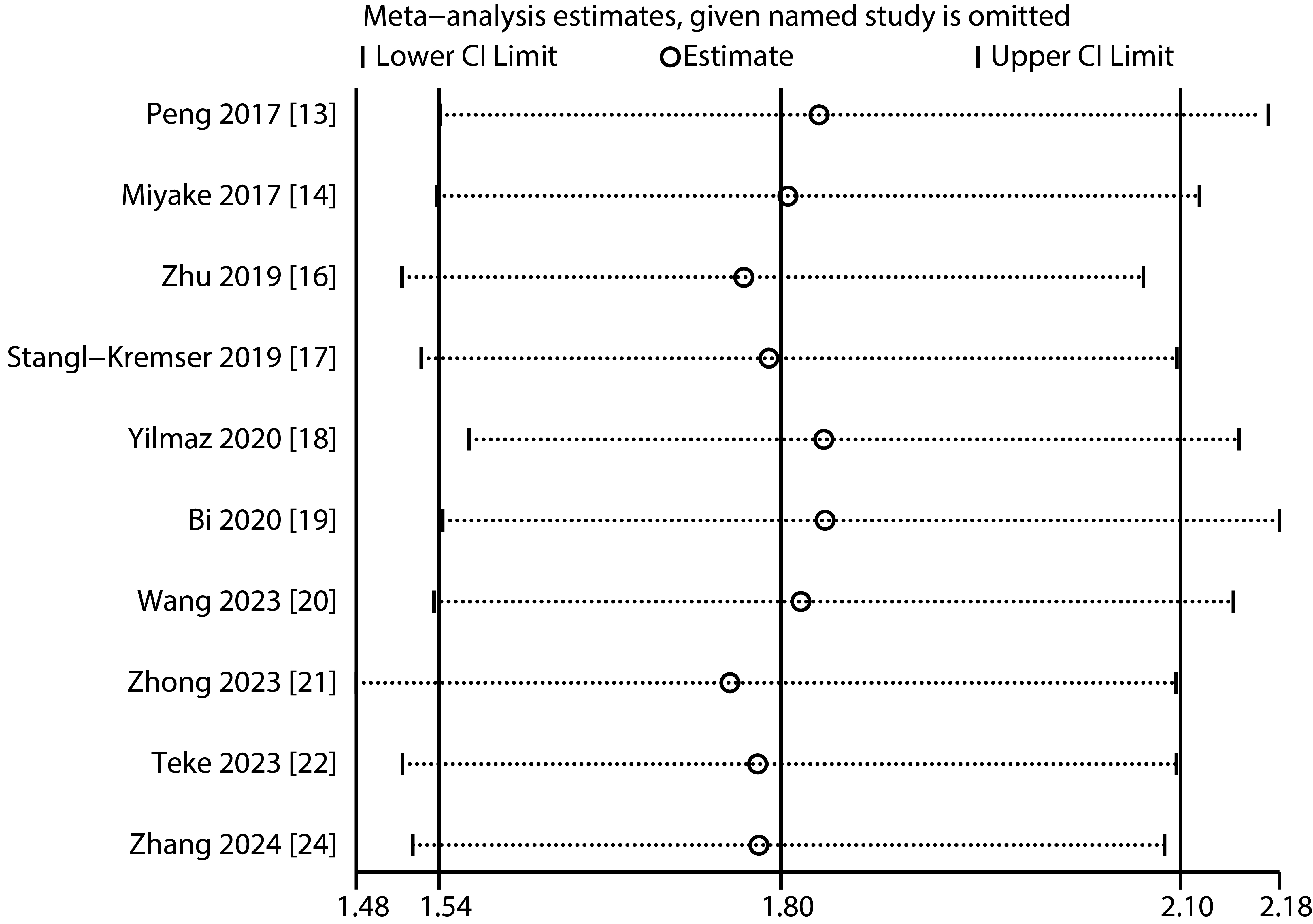

Supplement: Supplementary file 1 [file Image1.jpeg]

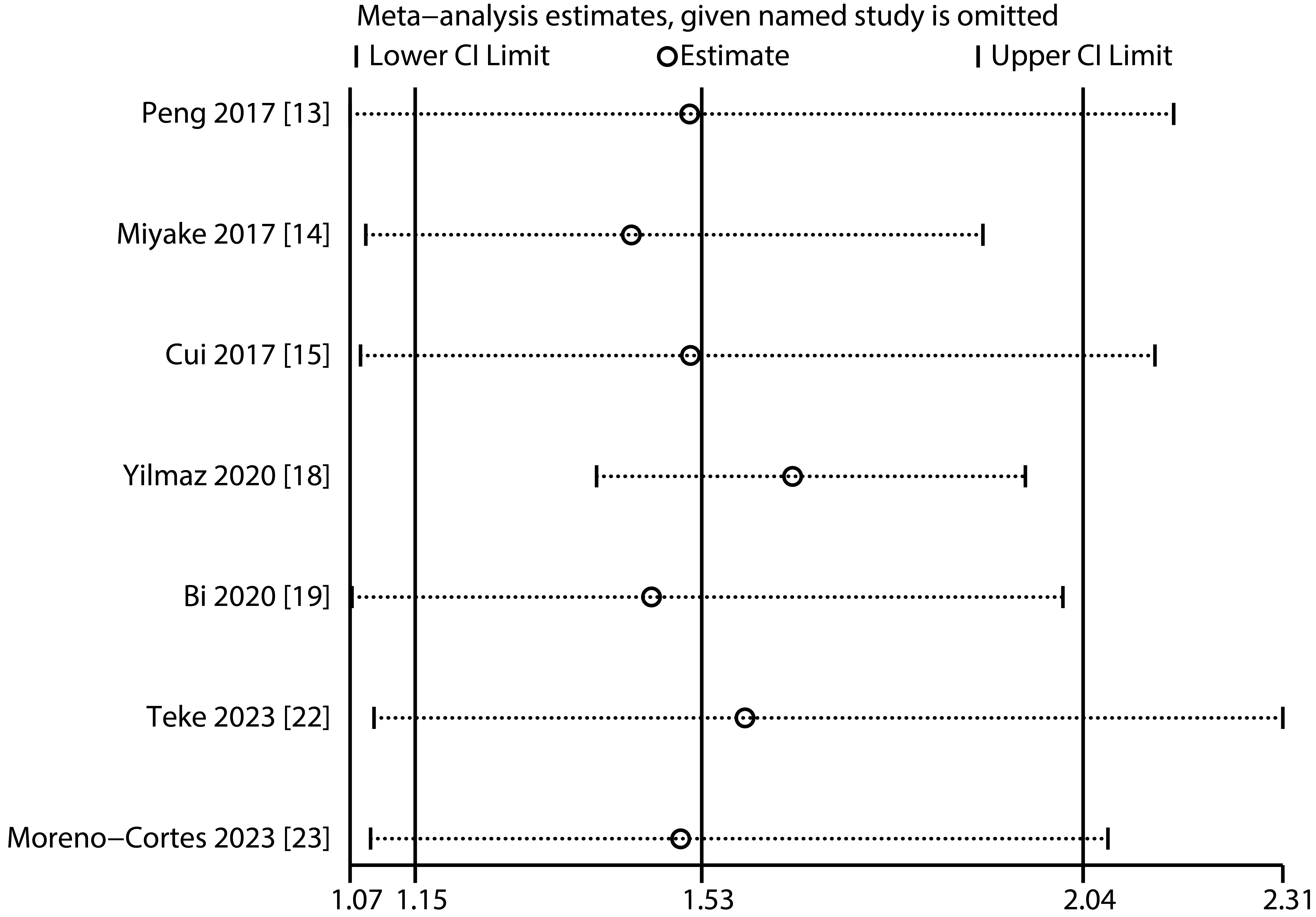

Supplement: Supplementary file 2 [file Image2.jpeg]
